# Aligning HIV treatment and hypertension clinic visits and dispensing as a first step towards service delivery integration in South Africa

**DOI:** 10.1002/jia2.26444

**Published:** 2025-07-07

**Authors:** Oratile Mokgethi, Amy Huber, Idah Mokhele, Khumbo Shumba, Vinolia Ntjikelane, Sydney Rosen, Sophie Pascoe

**Affiliations:** ^1^ Health Economics and Epidemiology Research Office Faculty of Health Sciences University of the Witwatersrand Johannesburg South Africa; ^2^ Department of Global Health Boston University School of Public Health Boston Massachusetts USA

**Keywords:** differentiated service delivery, HIV co‐morbidity, hypertension, interaction burden, service integration, South Africa

## Abstract

**Introduction:**

Global and national guidelines recommend the integration of care for HIV and other chronic conditions to improve individual and public health outcomes. South Africa's differentiated service delivery (DSD) models extend beyond HIV care, relying on pickup points that also distribute hypertension (HTN) medications. We assessed the alignment between antiretroviral treatment (ART) and HTN medication collection visits and dispensing intervals as an indicator of integration progress.

**Methods:**

The AMBIT project conducted a SENTINEL survey across 18 public clinics in three South African districts between September 2022 and April 2023, enrolling adult clients ≥ 6 months on ART. We recruited up to 180 clients across each model of care: conventional care‐not DSD eligible (conventional‐not‐eligible); conventional care‐DSD eligible but not enrolled (conventional‐eligible); facility‐ (FAC‐PuP) and external (EX‐PuP) pickup points. Healthcare interaction data were extracted from paper and electronic sources for clients with a 12‐month observation period. We analysed both self‐reported alignment and actual visit data. We estimated the number and proportion of HTN visits aligned with ART dispensing. Log‐binomial regression estimated adjusted risk ratios (ARR) to assess the association with a higher visit burden (> 5 interactions).

**Results:**

Of 724 enrolled, 644 (90%) client records were successfully linked (76% female; median age 42; 15% Conventional‐not‐eligible; 17% Conventional‐eligible; 18% FAC‐PuP; 28% EX‐PuP). Among these, 85 (13%) with HTN (81 self‐reported, 4 from medical records), self‐reported 94% and 95% aligned facility visits and medication pickups, respectively. Visit data was retrieved for self‐reported HTN diagnoses. Of 477 visits for HIV/HTN comorbid clients, 83% (395) dispensed both ART and HTN medication, and 97% had aligned dispensing durations (Conventional‐not‐eligible 97%, Conventional‐eligible 95%, FAC‐PuP 98%, EX‐PuP 100%). Comorbid clients had a similar visit burden to ART‐only clients (ARR 1.05, 95% CI: 0.80−1.39). FAC‐PuP (ARR 0.55, 95% CI: 0.40−0.78) and EX‐PuP (ARR 0.75, 95% CI: 0.57−0.98) clients were less likely than Conventional‐E clients to have high annual visit burden.

**Conclusions:**

Aligning medication visits and dispensing for HIV and other chronic diseases marks an initial step towards integrated service delivery. Our results demonstrate achievable medication visit alignment without increased visit burden for comorbid clients and those in DSD models, suggesting that HIV‐HTN integration is feasible within DSD models, matching client preferences for comprehensive care.

## 1 INTRODUCTION

The long‐term success of national HIV treatment programmes in sub‐Saharan Africa will depend in part on their ability to integrate care for the growing burden of non‐communicable diseases (NCDs) among people living with HIV (PLHIV) [1, 2]. As this population ages and lives longer due to effective antiretroviral treatment (ART), they become increasingly susceptible to developing NCDs like hypertension (HTN) and diabetes [3, 4]. This rising comorbidity poses new challenges for health systems and care delivery models originally designed to manage HIV vertically, as a single disease.

In recognition of this shift, global and national guidelines now emphasise the importance of integrating HIV treatment with the management of other chronic diseases [5, 6]. South Africa, with over 5.5 million PLHIV receiving ART, has been at the forefront of this effort [7, 8]. The Centralized Chronic Medicine Distribution and Dispensing (CCMDD) programme operates in eight of the country's nine provinces, using public clinics and private couriers to deliver chronic medications including HIV and HTN. CCMDD supports South Africa's main low‐intensity differentiated models of care (DMOCs, also called differentiated service delivery [DSD] models) [9]. Among other goals, these models aim to align the timing of ART and HTN medication delivery to minimise the number of interactions with the healthcare system required for clients with multiple conditions.

Alignment of visits and dispensing represents an important step towards achieving true integrated service delivery for HIV and comorbid conditions. Although simultaneous dispensing of ART and HTN medications is intended through CCMDD, the extent to which care for HIV and common NCDs is aligned in terms of clinic visit schedules and actual medication dispensing intervals remains unclear. This study examined the alignment of care for clients receiving medication for HIV and HTN and investigated factors associated with a high visit burden for clients.

## 2 METHODS

### 2.1 Study setting, population and data

In South Africa, non‐pregnant ART clients with documented viral suppression, ≥ 6 months’ experience on treatment and control of co‐morbidities were eligible for one of three “repeat prescription strategies” or DSD models during the study period. These included facility‐based medication pickup points (Fac‐PuP), community‐based or external medication pickup points (Ex‐PuP), and, less commonly, adherence clubs [10]. ART clients remained in conventional care if they were ineligible for DSD, declined enrolment or were not offered enrolment due to facility or provider preferences or limitations. Dispensing durations were 1–2 months in conventional care and 2–3 months in DSD models.

The multi‐component SENTINEL 2.0 study was conducted between September 2022 and April 2023 at 18 public healthcare facilities in three South African districts: King Cetshwayo in KwaZulu‐Natal Province, Ehlanzeni in Mpumalanga Province and West Rand in Gauteng Province. The sites represented a mix of ART patient volumes and included both urban and rural locations [11]. All followed South Africa's national guidelines for HIV care and for DSD [12].

At each study site, we first recruited up to 10 ART clients in each of four categories: (1) not eligible for DSD and remaining in conventional care (conventional‐not‐eligible); (2) eligible for DSD but remaining in conventional care (conventional‐eligible); (3) enrolled in Fac‐PuP; and (4) enrolled in Ex‐PuP. (We did not find enough clients enrolled in adherence clubs to include this model in the study.) Participants were approached sequentially as they arrived at the study sites, screened for study eligibility and invited to participate. Those who provided written informed consent were enrolled in the study.

We then administered a structured questionnaire that included questions about access to and experience with HIV treatment and comorbidity care during routine visits (Supporting information Sentinel survey) [11]. We conducted a manual review of clients’ medical records, including paper files, CCMDD prescription sheets and the CCMDD database for chronic disease medications [13], to capture data on clinic visits, off‐site medication collection visits, medications dispensed (with HTN medication dispensing not consistently documented), and dispensing intervals and dates. We defined a 12‐month observation period for each study participant, starting from the most recent visit captured in our dataset and looking back over the preceding 12 months.

### 2.2 Analysis and outcomes

The objective of this analysis was to estimate visit alignment, defined as services received on the same day from the same facility for clients receiving medication for both HIV and HTN, among those who self‐reported an HTN diagnosis during the survey, stratified by DSD model. Alignment was determined using the dates the services were received, as our data sources did not include time stamps or specific location information within facilities. Co‐morbidity status was classified into three subgroups based on self‐reported survey data: (1) ART only (no additional conditions); (2) ART with HTN treatment (with or without other treatments); and (3) ART with other treatment(s) but no HTN treatment.

We assessed visit alignment by first determining the total number of facility visits within the observation period. For clients with a documented HTN diagnosis date, we included all facility visits; for those without a documented HTN diagnosis date, we included facility visits from their first recorded dispensing of HTN medication onwards. We next identified aligned visits and calculated the proportion of aligned visits among total visits. Finally, for the alignment of medication dispensing durations where the number of months was documented for both conditions, we identified visits where the duration dispensed for ART matched the duration of HTN treatment, calculating the proportion of visits with the same dispensing duration among aligned visits.

For each participant, the healthcare interaction burden was defined as the sum of the number of facility‐based clinical visits, facility‐based medication collection visits and external medication collections during the observation period. We created a binary variable using the sample's median number of 5 interactions per year within the full sample as the cut‐off to distinguish between low (≤ 5 visits/year) and high interaction burden (> 5 visits/year). We used a log‐binomial regression with binomial errors to investigate factors associated with high healthcare interaction burden and estimate crude and adjusted relative risks. This method used a logarithmic link function and robust Poisson regression and adjusting for variables known to influence healthcare interaction and alter the crude risk ratios by more than 10% (age, sex, duration on ART, marital status and location) [14, 15, 16]. Clients who transitioned between care models were excluded from the analysis as they represented a mix of care models over time.

## 3 RESULTS

### 3.1 Enrolment and classification of enrolment in the study

**Figure 1:**
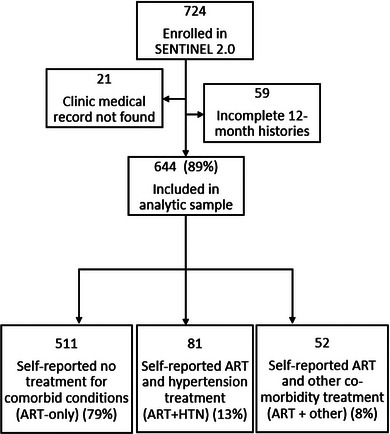
**Study population flow diagram: Enrolment and classification of participants in the Sentinel 2.0 survey examining alignment of HIV treatment with HTN treatment**. Abbreviations: ART, antiretroviral therapy; HTN, hypertension.

Enrolment in the SENTINEL 2.0 survey is illustrated in Figure 1. Of the 644 participants in the analytic sample, 132/644 (21%) self‐reported having conditions in addition to HIV. HTN was most common, with 81/644 clients (13%) self‐reporting HTN treatment. Of these HTN clients, 16/81 also reported receiving treatment for another condition in addition to HIV and HTN; they were included in the ART+HTN subgroup. The remaining 51/132 clients reported other conditions like diabetes without HTN. We also identified 21 clients with some evidence of HTN treatment in medical records but no self‐report. Upon review, 4/21 had both a diagnosis date and consistent HTN treatment and were included in the ART+HTN subgroup, bringing the final ART+HTN subgroup sample to 85. The other 17/21 were kept in their respective groups: ART‐only or ART+ other due to potential acute or inconsistent medication use. The analytic cohort is described in Table 1.

**Table 1:** Demographic, clinical and care integration characteristics of people living with HIV receiving treatment in three South African districts, 2022–2023

Characteristic (*n*, %)	ART only	ART and HTN treatment	ART and other co‐morbidity treatment^a^	Total
*N*	508	85	51	644
**Demographic, social and economic characteristics**				
Sex (female)	381 (75%)	69 (81%)	39 (76%)	489 (76%)
Age (years)				
Median (IQR)	40 (34, 47)	51 (42, 60)	47 (38, 52)	42 (35, 49)
18−24	27 (5%)	0 (0%)	0 (0%)	27 (4%)
25−34	149 (29%)	4 (5%)	8 (15%)	161 (25%)
35−49	265 (53%)	39 (46%)	27 (53%)	331 (52%)
50+	67 (13%)	42 (49%)	16 (31%)	125 (19%)
Marital status				
Never married (currently single)	84 (17%)	16 (19%)	14 (27%)	114 (18%)
Never married (currently in a relationship)	299 (59%)	35 (41%)	25 (49%)	359 (56%)
Married	91 (18%)	20 (24%)	10 (20%)	121 (19%)
Divorced/separated/widowed	34 (6%)	14 (16%)	2 (4%)	50 (8%)
Model of care for HIV treatment				
Conventional‐not‐eligible	64 (13%)	23 (28%)	22 (42%)	109 (17%)
Conventional‐eligible	80 (16%)	13 (14%)	4 (8%)	97 (15%)
Fac‐PuP	93 (18%)	15 (19%)	7 (14%)	115 (18%)
Ex‐PuP	159 (31%)	14 (17%)	7 (14%)	180 (28%)
Transitioned	112 (22%)	18 (22%)	11 (22%)	143 (22%)
Duration on ART at time of survey (years)				
Median (IQR)	6 (3, 10)	7 (4, 11)	8 (3, 11)	7 (3, 10)
6 months−1 year	43 (8%)	1 (1%)	4 (8%)	48 (7%)
2−4	156 (31%)	25 (29%)	12 (24%)	193 (31%)
5−9	189 (37%)	28 (34%)	18 (35%)	235 (36%)
10+	120 (24%)	31 (36%)	17 (33%)	168 (26%)
Time on HTN^b^ (with diagnosis date)				
Median (IQR)	−	4 (2,7)	−	
District				
West Rand	164 (32%)	47 (55%)	13 (26%)	224 (35%)
Ehlanzeni	197 (39%)	23 (27%)	20 (39%)	240 (37%)
King Cetshwayo	147 (29%)	15 (18%)	18 (35%)	180 (28%)
Location				
Rural	262 (52%)	33 (42%)	26 (51%)	324 (50%)
Urban	246 (48%)	49 (58%)	26 (49%)	320 (50%)
**Self‐reported clinical characteristics and alignment of care**				
Self‐reported co‐morbid condition(s)^c^	0	81	51	132
Hypertension	0	81 (100%)	0 (0%)	81 (61%)
Diabetes	0	6 (7%)	11 (23%)	17 (13%)
Asthma	0	3 (2%)	6 (4%)	9 (7%)
TB	0	0 (0%)	7 (14%)	7 (5%)
Mental health	0	2 (2%)	7 (14%)	9 (7%)
Other	0	5 (6%)	23 (45%)	28 (19%)
Are you able to combine your ART visits with your additional diseases?^d^				
Always	n.a.	76 (94%)	44 (86%)	120 (91%)
Very often	n.a.	3 (4%)	0 (0%)	3 (2%)
Sometimes	n.a.	0 (0%)	1 (2%)	1 (1%)
Rarely/never	n.a.	2 (2%)	6 (12%)	8 (6%)
Can you pick up your medication alongside your HIV medications?				
Always	n.a.	77 (95%)	43 (85%)	120 (90%)
Very often	n.a.	1 (1%)	0 (0%)	1 (1%)
Sometimes	n.a.	0 (0%)	1 (2%)	1 (1%)
Rarely/never	n.a.	3 (4%)	7 (13%)	11 (8%)

Abbreviations: ART, antiretroviral therapy; DSD, differentiated service delivery; Ex‐PuP, external medication pickup points; Fac‐PuP, facility‐based medication pickup points; HTN, hypertension; IQR, interquartile range; n.a., not applicable; TB, tuberculosis.

^a^ Other treatment includes chronic conditions, communicable diseases and opportunistic infections.

^b^ 55/85 individuals with diagnosis date.

^c^ Individuals can fall into more than one category.

^d^ Care integration measures (ability to combine visits and pick up medications) were only assessed for participants reporting co‐morbid conditions.

### 3.2 Socio‐demographic and clinical characteristics of people living with HIV in the study

Clients receiving ART and HTN treatment were found in all DSD models of care as shown in Table 1. The ART‐only subgroup was somewhat younger than the ART+HTN subgroup (median age 40 vs. 51 years). A higher proportion of clients in the ART+HTN subgroup resided in urban areas (58% vs. 48% for ART‐only clients). Nearly, all (76/81; 94%) clients receiving both ART and HTN treatment reported that they always combine their HIV visits with HTN care, and 95% (77/81) reported that they always pick up their HTN medication alongside their HIV medications.

### 3.3 Alignment of care for clients receiving medication for HIV and HTN

The 85 participants receiving ART+HTN treatment had a total of 477 facility‐based medication dispensing visits during the 12‐month observation period. Across all model of care categories, the majority (395 visits, 83% overall) of facility visits for clients receiving both ART and HTN medication were aligned (ART and HTN medications were dispensed during a single visit): 86% for conventional‐not‐eligible, 82% for conventional‐eligible, 70% for Fac‐PuP, 84% for Ex‐PuP and 86% for transitioned. For visits at which medications were not dispensed for all conditions, clients most commonly received only ART (8% of visits for conventional‐not‐eligible, 13% for conventional‐eligible, 20% for Fac‐PuP, 10% for Ex‐PuP and 5% for transitioned). Very few visits resulted in HTN medication being dispensed alone or in no medications being dispensed. Duration of dispensing (number of months dispensed at a visit) for ART and HTN medications was aligned in 97% (319/329) of client visits with complete dispensing duration documentation across the different models of care. Dispensing alignment was 97% for conventional‐not‐eligible, 95% for conventional‐eligible, transition clients (98%), Fac‐PuP clients (98%) and 100% for Ex‐PuP.

### 3.4 Factors associated with a high visit burden for all clients in the study

The median visit burden (annual number of healthcare interactions per year) for participants (*N* = 508) in our cohort was 5.0. By model of care, participants in the conventional‐not‐eligible had a median visit burden of 7.0 and a median medication dispensing of 1 month, followed by conventional‐eligible with 6.0 interactions per year and a median dispensing of 2 months. Both Fac‐PuP and Ex‐PuP had a median of 4.0 interactions per year, with median medication dispensing of 2 months.

**Figure 2:**
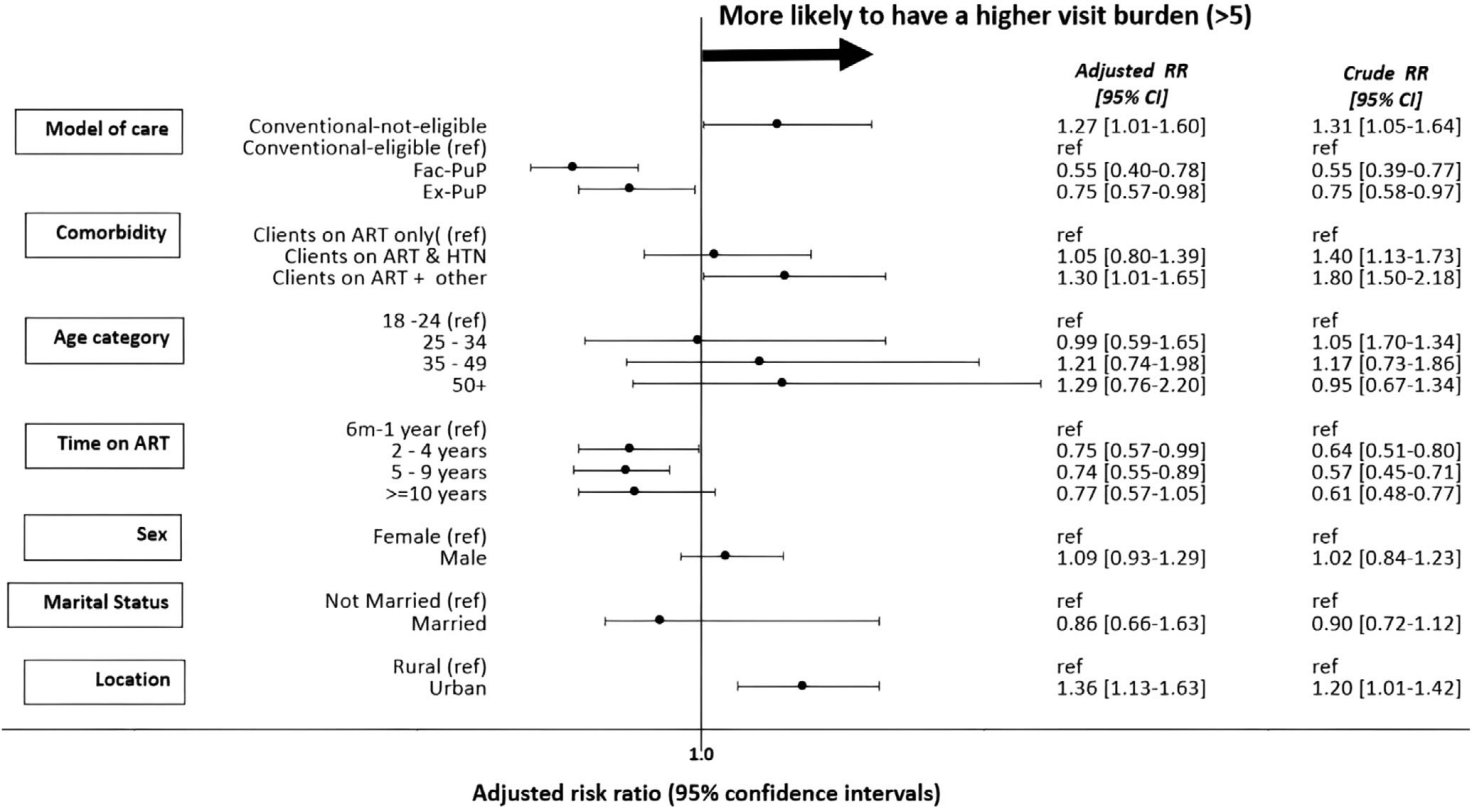
**Forest plot presenting the crude and adjusted risk ratios for high interaction burden (> 5 interactions) among differentiated service delivery model and comorbidity status (*n* = 508), adjusting for age, sex, duration on ART, marital status and location of facility**. *For this analysis, we included 508 clients; we excluded the 136 clients in the Transition category. Abbreviations: ART, antiretroviral therapy; CI, confidence interval; HTN, hypertension; PuP, pick‐up point; RR, risk ratio; Ref, reference.

Figure 2 illustrates associations between client characteristics and the risk of having a high visit burden. Clients in conventional care who were not eligible for DSD had a significantly higher visit burden than did those in conventional care who were eligible for DSD (adjusted relative risk 1.27, 95% CI 1.01−1.60). Both DSD models showed significantly lower visit burdens than did conventional‐eligible (Fac‐PuP 0.55, 0.40−0.78; Ex‐PuP 0.75, 0.57−0.98). There was no significant difference in the risk of high visit burden between ART‐only and ART+HTN clients, but ART+ other clients had a significantly higher visit burden (1.30, 1.01−1.65). Clients on ART for 2–4 years (0.75, 0.57−0.99) and 5–9 years (0.74, 0.55−0.99) experienced a lower visit burden compared to those on treatment for 6 months to 1 year.

## 4 DISCUSSION

In this study of the alignment of HIV and HTN care in South Africa, we found that a large majority of ART and HTN medication refill visits and dispensing durations were aligned across all models of care. To our surprise, we observed no significant difference in visit burden between clients receiving only ART and those receiving ART and HTN medication. We had expected that the need to provide care for multiple chronic conditions would increase logistical demands (visits, dispensing events), but the DSD models employed in South Africa appear to have achieved a high degree of visit and dispensing alignment without adding to the overall burden for affected clients or facilities.

The level of ART‐NCD visit alignment reported in our study is much higher than the 60% previously reported in an earlier study conducted in the Western Cape Province in 2020 in a population with similar characteristics [17]. Our results may reflect differences among the provinces—Western Cape does not utilise the same dispensing system or models of care as the rest of South Africa—or may indicate progress towards greater alignment in the past 4 years.

The data utilised for this study allowed us to document the alignment of care within a single clinic visit, but it did not indicate the total number of healthcare providers seen during the course of that visit. Even clients with perfect visit and dispensing alignment in our study may have been required to see multiple providers in different parts of the clinic. Concern also remains about the lack of integration of tools and systems recording this information. TIER.Net, South Africa's HIV electronic medical record, captures only HIV and TB‐related services making it difficult to track co‐morbid clients. Integrating records across different conditions is essential to fully understanding integrated care.

The main limitations of our study were reliance on self‐report to identify treatment of co‐morbidities and on potentially incomplete medical record data to evaluate alignment. It is likely that some clients receiving treatment for conditions other than HIV did not disclose this in the survey, potentially reducing observed differences between the ART‐only and ART+HTN categories. Although we conducted a manual record review, the clinical stationery used did not have structured fields for non‐HIV chronic conditions, potentially leading to missing information about NCD care, in particular for HTN dispensing duration. If HTN‐related interactions were missed, our study would overestimate the actual level of alignment achieved. Finally, the small sample size for clients with both HIV and HTN and lack of HTN clinical outcome data constrained our ability to evaluate the impact of the alignment observed.

South Africa's journey towards integrated chronic care delivery offers valuable insights for health systems globally. Through the Integrated Chronic Disease Management model [18], the country has implemented practical strategies including facility reorganisation, population screening, standardised treatment protocols and patient empowerment programmes. South Africa successfully merged a previously vertical HIV treatment programme into a broader chronic disease care model that allowed the alignment of visits and dispensing reported above, a step that many other countries have not yet taken.

## 5 CONCLUSIONS

This study demonstrates that successfully aligning treatment visits and medication dispensing for clients with multiple chronic conditions may be achievable in both conventional and differentiated care settings and lead to a reduction in the burden of care‐seeking for clients with multiple conditions.

## COMPETING INTERESTS

The authors declare that they have no competing interests.

## AUTHORS’ CONTRIBUTIONS

NOM, AH, IM, KS, SP and SR conceptualised the study. AH and VN oversaw data collection. OM did data analysis and drafted the manuscript with inputs from AH, IM, KS, VN and SR. All authors contributed to reviewing and editing the manuscript for submission.

## FUNDING

Funding for the study was provided by the Bill & Melinda Gates Foundation through INV‐037138 to Wits Health Consortium. The funders had no role in study design, data collection and analysis, decision to publish or preparation of the manuscript.

## Supporting information




**Supporting information**: Sentinel survey.

## Data Availability

Data available on request from the authors.
