# Supplementary material for: Aligning HIV treatment and hypertension clinic visits and dispensing as a first step towards service delivery integration in South Africa
Source: J Int AIDS Soc. 2025 Jul 7;28(Suppl 3):e26444. doi: 10.1002/jia2.26444 (PMC12232478; doi:10.1002/jia2.26444)
Supplement: Supplementary file 1 — Supporting information: Sentinel survey. [file JIA2-28-e26444-s001.pdf]

Supporting Information: Sentinel survey

Screening number	Survey ID

PART B: SURVEY

V. Patient survey

Location within facility ______________________

Surveyor notes:

Introduction

Surveyor: Read the following statement. Please repeat the statement translated into the local language based on primary languages.

"Thank you for agreeing to participate in this survey. My name is ______. I will be asking you the questions. Most of the questions require that you select one of the options as your answer, although some questions you can select all the answers that apply. I will specify the options and instructions for you as I ask each question. If your answer is not one of the specified options please tell me and I will write your answer down. Please feel free to tell me whatever you are comfortable sharing. You should also remember that you do not have to share anything that you are not comfortable sharing and that you can stop this interview at any time without any risk to your rights or treatment and care. There are no right or wrong answers, so please be honest and help us to understand what is true for you and your community. Are you ready to begin?

Surveyor: "I'm going to start by asking you some basic questions about who you are, where you live, and your education and employment."

Q#	QUESTION	RESPONSES
**Respondent demographics and socio-economic status**		
1.	What is your nationality/country of origin?	1= South Africa<br>2= Botswana<br>3= Lesotho<br>4= Mozambique<br>5= Malawi<br>6= Namibia<br>7= Swaziland<br>8= Zambia<br>9= Zimbabwe<br>10=Tanzania<br>11=Burundi<br>12= Other African country (specify)<br>13= Other (specify)
2.	If you are not South African, how long have you lived in South Africa?	1= < 1 yr<br>2= 1-2 years

Q#	QUESTION	RESPONSES
		3= 2-5 years<br>4= >5 years<br>5= Seasonal work (Only work in South Africa during certain times of the year)
3.	What is your marital status?	1= Never married<br>2= Married (customary/traditional or legal/civil)<br>3= Divorced<br>4= Separated<br>5= Widowed
4.	Is there someone who you have a relationship with and who you call your partner?	1= No<br>2= Yes
5.	Do you currently live with your husband/wife or your partner?	1= No<br>2= Yes, married or living together
6.	Do you think of the house you currently live in as your main house?	1= Yes<br>2= No, my main house is somewhere else in South Africa<br>3= No, my main house is in another country
7.	Do you know how to read and write?	1= No<br>2= Yes – read and write<br>3= Yes – read only
8.	What was the highest level of school that you completed?	1 = No schooling<br>2= Primary<br>3= Secondary<br>4= Certificate/Diploma/ Post-secondary<br>5= Graduate degree
9.	What is your occupation?	1= Farming (my own or my family's farm)<br>2= Farm worker (someone else's farm)<br>3= Domestic worker or carer (paid)<br>4= Informal sector job (not farming or domestic) (e.g. trader, day service provider)<br>5= Formal sector job (salaried)<br>6= Household work and/or childcare (my own house, not paid)<br>7= Unemployed but looking for work<br>8= Student or trainee<br>9= Retired<br>10= Other (specify)<br>11= Self-employed/own business<br>12= Unemployed but not looking for work
10.	Where do you get MOST of your money from?	1= Salary, business or job (formal or informal sector)<br>2= Government social grant<br>3= Spouse/partner<br>4= Parents/relatives<br>5= Friends<br>6= Other (specify)
11.	Do you or the people in your household go without food often, sometimes, seldom, never?	1= Never<br>2= Seldom<br>3= Sometimes<br>4= Often

Q#	QUESTION	RESPONSES
12.	Do you or does anybody in your household, currently receive any support or grant from the government? Tick all that apply)	0= No<br>1= Child grant<br>2= Partial disability / illness grant<br>3= Pension grant<br>4= Disability grant<br>5= Unemployment grant/ UIF<br>6= Other (specify)<br>7= COVID social relief grant
13.	If a person in your household became ill and 100 Rands was needed for treatment or medicines, would you say it would be very easy, easy, difficult, or very difficult to find the money?	1= Very difficult<br>2= Difficult<br>3= Easy<br>4= Very easy
**Healthcare access and cost**		
14.	How long have you been taking ART?	Months/years
15.	On average, how many months of ART medication do you receive at a time?	Number
16.	Which health care services are you routinely receiving at this facility, in addition to HIV care? (Tick all that apply)	0= None<br>1= TB treatment<br>2= Diabetes<br>3= Hypertension<br>4= Asthma<br>5= Mental health<br>6= Malaria<br>7= Family planning<br>8= Antenatal care<br>9= Child health care<br>10= TB preventative therapy (TPT)<br>11= Other (specify)
17.	Do you receive treatment for any of these conditions/diseases or as part of the health care services you are routinely receiving at this facility?	0= No<br>1= Yes
18.	If yes, what conditions/diseases do you receive treatment for?	1= TB treatment<br>2= Diabetes treatment<br>3= Hypertension treatment<br>4= Asthma treatment<br>5= Mental health treatment<br>6= Malaria<br>7= Other
19.	Are you able to combine your HIV visits with the visits for the other health care services?	0= Always<br>1= Very often<br>2= Sometimes<br>3= Rarely<br>4= Never
	a. TB	
	b. Diabetes	
	c. Hypertension	
	d. Asthma	
	e. Mental health	
	f. Malaria	

Q#	QUESTION	RESPONSES
	g. Family Planning	
	h. Antenatal care	
	i. Child health care	
	j. TB preventative therapy (TPT)	
	k. Other (specify)	
20.	Are you able to collect your medication for these other diseases/conditions at the same time as you collect your HIV medication?	0= Always<br>1= Very often<br>2= Sometimes<br>3= Rarely<br>4= Never
	a. TB	
	b. Diabetes	
	c. Hypertension	
	d. Asthma	
	e. Mental health	
	f. Malaria	
	g. TB preventative therapy (TPT)	
